# Quantitative Analysis of the Effect of Cancer Invasiveness and Collagen Concentration on 3D Matrix Remodeling

**DOI:** 10.1371/journal.pone.0024891

**Published:** 2011-09-27

**Authors:** Dewi Harjanto, Joseph S. Maffei, Muhammad H. Zaman

**Affiliations:** Department of Biomedical Engineering, Boston University, Boston, Massachusetts, United States of America; Instituto de Engenharia Biomédica, University of Porto, Portugal

## Abstract

Extracellular matrix (ECM) remodeling is a key component of cell migration and tumor metastasis, and has been associated with cancer progression. Despite the importance of matrix remodeling, systematic and quantitative studies on the process have largely been lacking. Furthermore, it remains unclear if the disrupted tensional homeostasis characteristic of malignancy is due to initially altered ECM and tissue properties, or to the alteration of the tissue by tumor cells. To explore these questions, we studied matrix remodeling by two different prostate cancer cell lines in a three-dimensional collagen system. Over one week, we monitored structural changes in gels of varying collagen content using confocal reflection microscopy and quantitative image analysis, tracking metrics of fibril fraction, pore size, and fiber length and diameter. Gels that were seeded with no cells (control), LNCaP cells, and DU-145 cells were quantitatively compared. Gels with higher collagen content initially had smaller pore sizes and higher fibril fractions, as expected. However, over time, LNCaP- and DU-145-populated matrices showed different structural properties compared both to each other and to the control gels, with LNCaP cells appearing to favor microenvironments with lower collagen fiber fractions and larger pores than DU-145 cells. We posit that the DU-145 cells' preference for denser matrices is due to their higher invasiveness and proteolytic capabilities. Inhibition of matrix proteases resulted in reduced fibril fractions for high concentration gels seeded with either cell type, supporting our hypothesis. Our novel quantitative results probe the dynamics of gel remodeling in three dimensions and suggest that prostate cancer cells remodel their ECM in a synergistic manner that is dependent on both initial matrix properties as well as their invasiveness.

## Introduction

Most cancers, including prostate cancer, only become fatal once cancer cells have metastasized to distant sites [1]. Cancer metastasis involves the migration of cells from the original tumor mass through the basement membrane and loose connective tissue into the blood or lymphatic system. Tumor cells then extravasate from the circulatory system to find their way to new tissues. During metastasis, cells interact with the extracellular matrix (ECM), a complex network of glycosaminoglycans, adhesion proteins, and structural fibers, such as collagen. Many properties of the ECM, including matrix structure [2], mechanics [3], and dimensionality [4], have been shown to impact cell behavior. Cells in vivo are usually surrounded on all sides by ECM, showing tissue-specific mechanical and structural properties. Consequently, it is important to study cells in tunable three-dimensional (3D) systems, which better mimic physiological conditions than flat, highly rigid substrates.

A critical step during in vivo cell migration, invasion, and metastasis is matrix remodeling. Matrix proteolysis is especially important for cells seeded in 3D since they are more likely to be sterically obstructed from moving than cells on planar substrates. Accordingly, it has been shown that inhibiting matrix metalloproteases (MMPs) reduces cell speed and persistence in 3D matrices but may not significantly alter tumor cell movement on 2D substrates [5], [6]. MMP expression also often increases during cancer progression [7], suggesting that advanced tumor cells more actively remodel the ECM to facilitate metastasis. MMP-2 and MMP-9 have been identified as important MMPs involved in metastatic prostate cancer [8]. Cells can also use their actomyosin machinery to pull on the matrix to align fibers [9]–[11].

While matrix remodeling is an important process, few studies have attempted to study it directly. Of particular interest is understanding how remodeling dynamically alters ECM structure. A tool that has been used to study ECM structure is confocal reflection microscopy (CRM). CRM collects light that is reflected by ECM fibers, allowing for the 3D structural reconstruction of the matrix. While recent work shows that CRM is blind to fibers oriented in the direction of the incident light, resulting in an overestimate network mesh size [12], CRM is still a widely utilized technique, providing informative data that can form the basis of comparative studies. CRM has been used previously to study collagen structure [13]–[16], fibrillogenesis [17], and how cells interact with the ECM [18], [19]. Unfortunately, many of the studies on cell-matrix interactions have been fairly qualitative, and they have yet to provide a direct comparison between the cell's invasiveness, initial collagen concentration, and/or the changes in structure over time.

In this paper, we aim to answer all of these questions quantitatively. We explore how matrix structural properties, as quantified by image analysis of CRM data, change over time to evaluate ECM remodeling by prostate cancer cells in a 3D collagen gel system, varying the concentration of collagen to determine the effect of the initial ECM structure and mechanics. The remodeling behavior for two prostate cancer cell lines, LNCaP and DU-145, are compared. LNCaP cells are a relatively slowly growing, androgen-sensitive cell line derived from a metastatic lesion to bone [20]. DU-145 cells are faster growing, androgen-insensitive, and derived from a metastatic lesion to the brain [21]. A number of studies have shown that DU-145 cells are more actively invasive than LNCaP cells [22]–[25]. From this work, we are able to gain quantitative insight into how two different tumor cell lines of varying invasiveness remodel the matrix in 3D environments. Our results provide a novel insight into the dynamics of cell-matrix interactions from the matrix perspective.

## Results

### Gels of varying collagen concentration show different mechanical and structural properties

**Figure 1:**
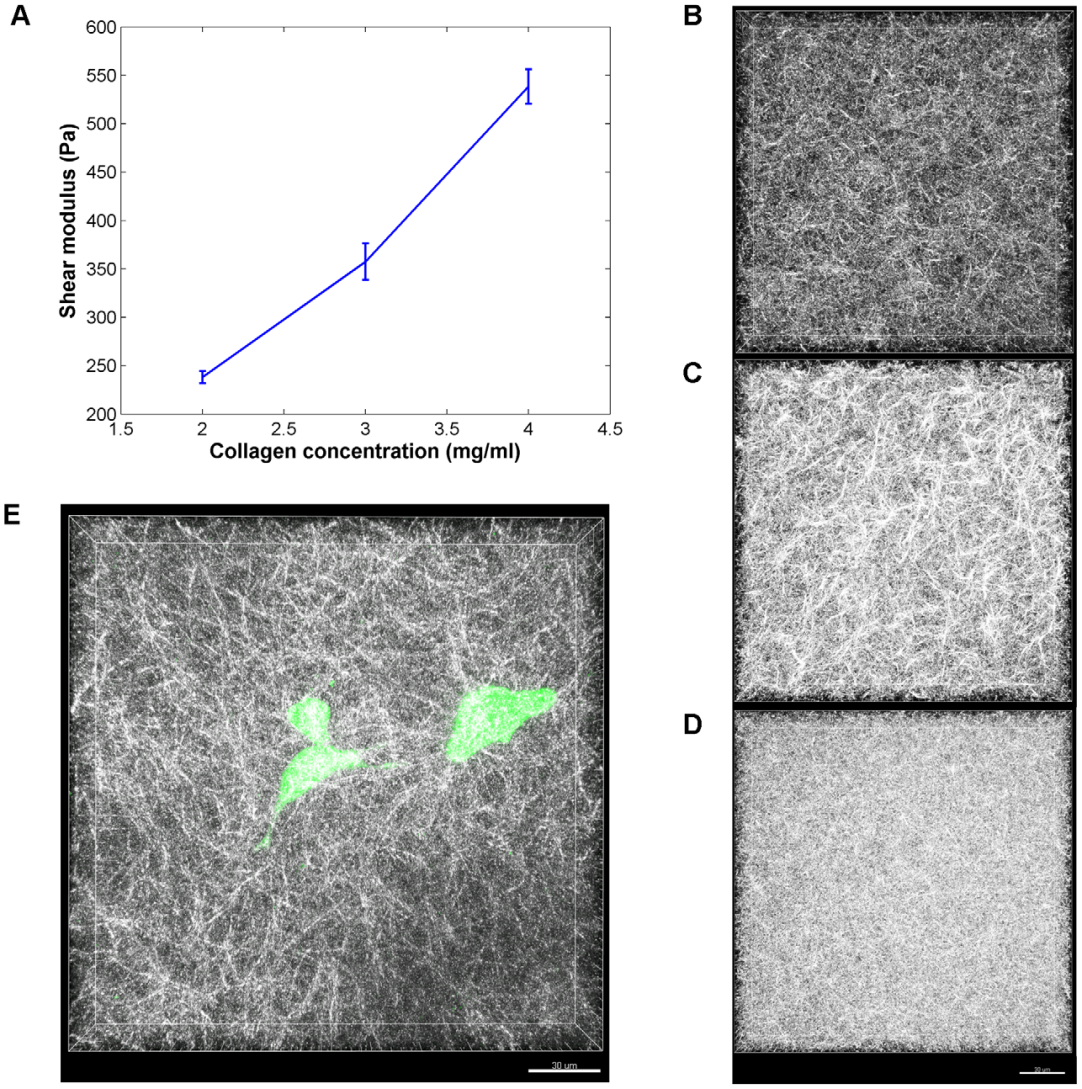
Extracellular matrix properties. (*A*) Shear modulus as a function of collagen concentration for gels without cells. Error bars: standard deviation. CRM images for gels without cells for (*B*) 2 mg/ml; (*C*) 3 mg/ml; and (*D*) 4 mg/ml. (*E*) CFM and CRM overlay of DU-145 cells (green) in 3 mg/ml collagen (white) 5 days after seeding. Scale bar for CRM images: 30 µm.

To obtain a baseline for comparison, 3D gels of varying collagen concentration (2, 3, or 4 mg/ml) that were not seeded with cells (identified as the “no cell” condition) were studied. Rheometry was performed to evaluate the initial stiffness of the gels. As expected, the shear modulus increased with collagen concentration, ranging from ∼200 to 550 Pa for 2 to 4 mg/ml gels (**Figure 1A**), yielding results comparable to those reported by other groups using a similar gelation technique [26].

**Figure 2:**
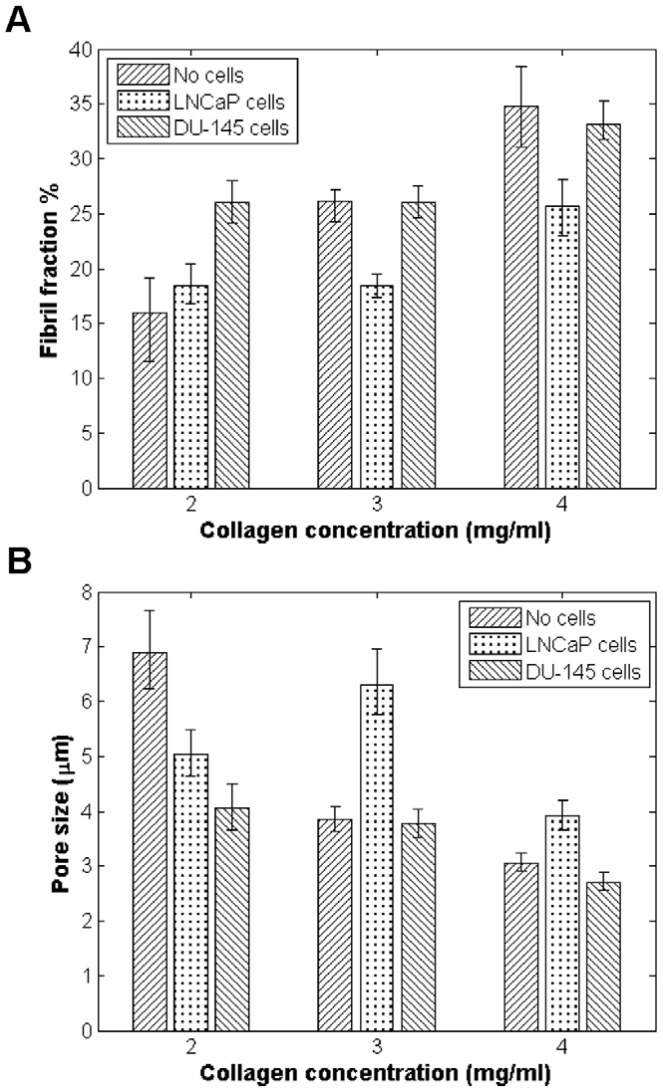
Comparison of structural parameters for gels without cells, with LNCaP cells, and with DU-145 cells. (*A*) Fibril fraction; and (*B*) pore size, one week after seeding. For cell-seeded gels, data from cellular regions are presented.

For structural analysis, CRM image stacks were acquired for the varying collagen gels 1, 3, 5, and 7 days after seeding. Representative CRM images of the three types of gels are shown in **Figure 1B–D**. In this study, the structural parameters of interest are the fraction of area collagen fibers occupy (referred to as “fibril fraction”), pore size, and fiber diameter and length. These parameters were selected as trackable, physiologically relevant metrics of matrix remodeling. Qualitatively, it is evident that higher concentration collagen results in gels that are more densely populated with fibers, resulting in smaller pore sizes. Quantitative image analysis corroborates these observations, as higher fibril fraction (**Figure 2A**) and smaller pore size (**Figure 2B**) is seen with higher collagen concentrations. Overall, there is no significant change over time for the fibril density and pore size (**Figure S1**) for the gels of each specific collagen concentration, as would be expected in the absence of matrix remodeling.

### LNCaP cells and DU-145 cells remodel the matrix in distinct manners dependent on the initial collagen concentration

Gels of varying collagen concentration (2, 3, 4 mg/ml) were then seeded with fluorescently-dyed prostate cancer cells. To visualize the stained cells, confocal fluorescence microscopy (CFM) was performed in conjunction with CRM. A representative image is shown in **Figure 1E**, showing that the cancer cells are clearly adhering to a 3D network of collagen fibers.

**Figure 3:**
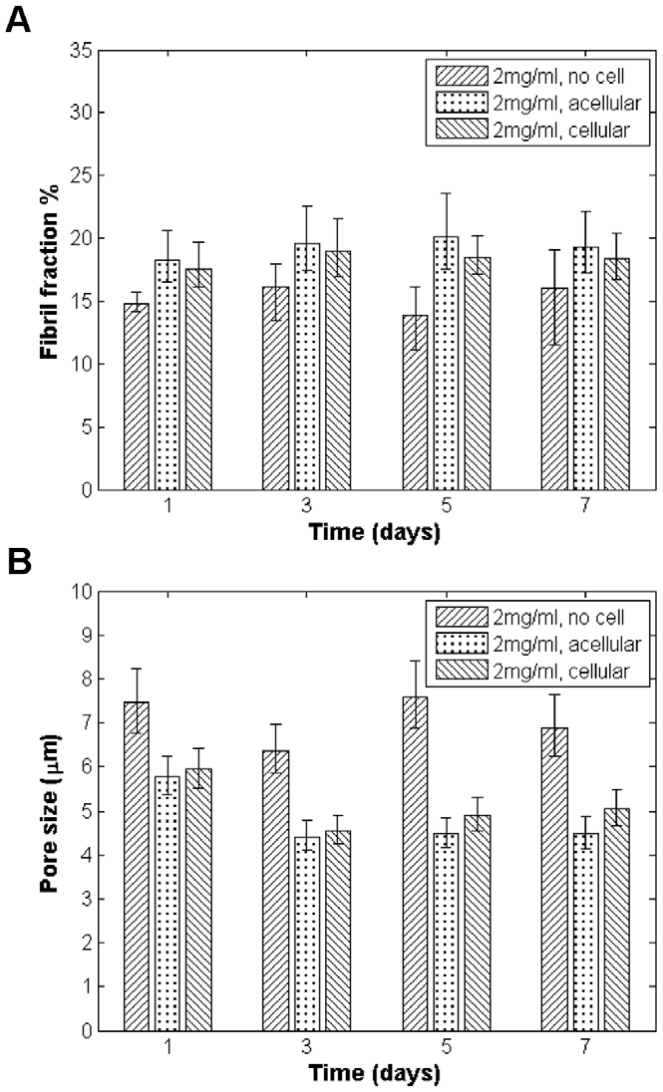
Comparison of structural parameters for 2 mg/ml gels seeded with LNCaP cells and without cells over time. (*A*) Fibril fraction; and (*B*) pore size, over time. Cellular regions of seeded gels contain cells; acellular regions do not.

As with the control gels, cell-seeded gels were imaged 1, 3, 5, and 7 days after plating to provide insight into the dynamics of matrix remodeling. To understand the extent that matrix remodeling occurs globally versus immediately local to cells, regions of the gel that at a given time point contained cells (identified as “cellular regions”) as well as regions that were not occupied by cells (identified as “acellular regions”) were imaged. Different regions were sampled between time points so it cannot be concluded that a given region was (or was not) occupied by cells over the course of the experiment as cells are motile. Nonetheless, it is interesting to see that while control gels show different structural properties compared to the seeded gels, there is no significant difference in fibril fraction and pore size from the acellular and cellular regions in gels seeded with LNCaP cells in 2 mg/ml collagen concentration over time (**Figure 3**). This is the case for both cell lines and for all gel concentrations at any given time point (data not shown). These results suggest that matrix remodeling occurs on a global scale, especially when the matrix is seeded with a relatively high density of cells, as is the case here.

**Figure 4:**
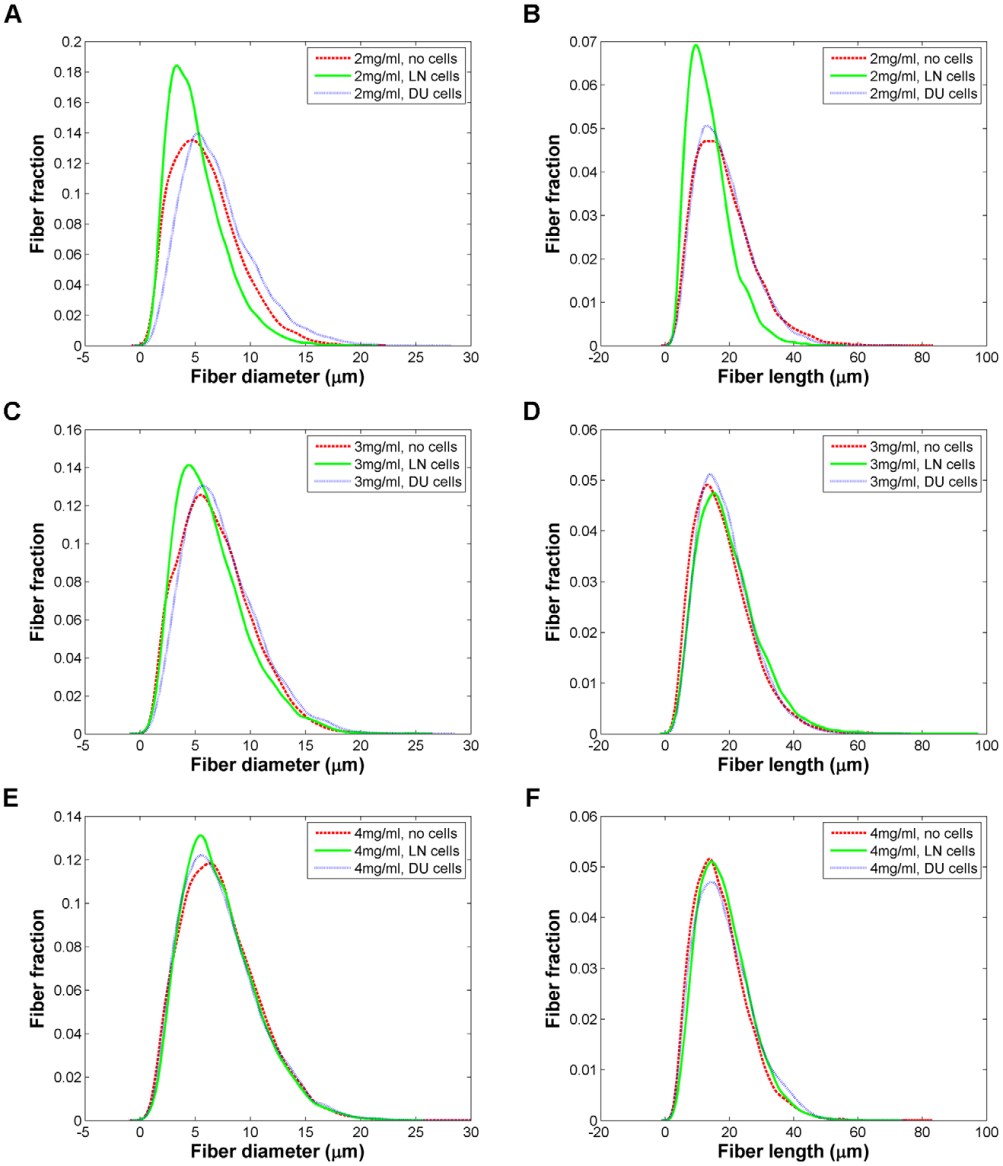
Comparison of fiber profiles for gels seeded without cells, with LNCaP cells, and with DU-145 cells. Fiber diameters for (*A*) 2 mg/ml; (*C*) 3 mg/ml; and (*E*) 4 mg/ml. Fiber lengths for (*B*) 2 mg/ml; (*D*) 3 mg/ml; and (*F*) 4 mg/ml. The y-axis indicates the decimal fraction of fibers with a given diameter or length, as is the case in the other fiber profile graphs (see also Figure S1C–F).

When LNCaP cells and DU-145 cells are seeded in collagen, there is a clear change in fibril fraction and pore size compared to the no cell condition (**Figure 2**). One week after seeding, compared to the control gels, both cell lines show higher collagen fibril content in 2 mg/ml gels and smaller pores. (Comparative data for the change in matrix properties for seeded gels over time can be seen in **Figure S2**.) DU-145 cells appear to deposit considerably more collagen than the LNCaP cells in 2 mg/ml gels. In higher collagen concentration gels, the behavior of the two cell lines diverges more. Compared to the control gels, the DU-145 cells do not appear to significantly change their environment, while the LNCaP-seeded 3 mg/ml and 4 mg/ml gels show significantly lower fibril fractions and correspondingly larger pores. The fiber length and diameter profiles for both cell lines are very comparable to what is observed in the no cell condition (**Figure 4**). It should be noted though that in the 2 mg/ml gels, more of a difference in these fiber properties was observed than at higher collagen densities, with LNCaP-seeded gels showing slightly narrower, shorter fibrils. LNCaP cells appear to favor environments with approximately ∼20% collagen occupancy, while DU-145 cells favor denser microenvironments with a fibril occupancy of ∼25–30% as the fibril fractions for all three collagen concentrations tested appear to converge to around these values over the course of experiments.

### LNCaP cells and DU-145 cells demonstrate different levels of proteolytic activity, and inhibition of this activity alters the matrix remodeling behavior observed

**Figure 5:**
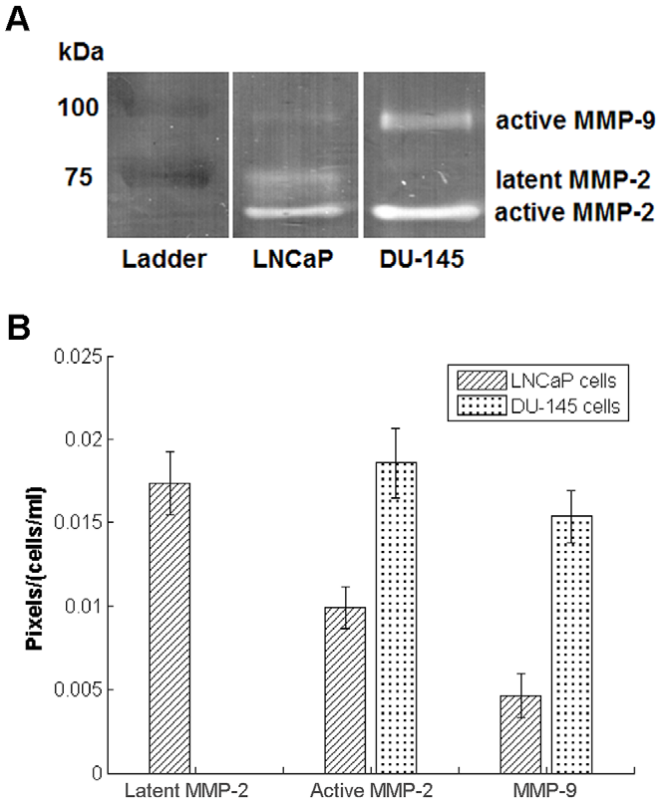
Assessment of proteolytic activity of LNCaP cells and DU-145 cells. (*A*) Gelatin zymogram; and (*B*) quantified data, with the pixel count of the area of proteolytic activity on the gel normalized by the cell density used in the experiment. Error bars represent standard error.

To directly assess the proteolytic activity and the invasiveness of the two different cell lines more directly, gel zymography was performed. As expected from previous studies [22]–[25], DU-145 cells appear to be much more invasive, showing higher levels of proteolysis by active MMP-2 and active MMP-9 than LNCaP cells (**Figure 5**). The increased invasiveness of DU-145 cells may account for their tolerance of more densely fibrous collagen microenvironments.

**Figure 6:**
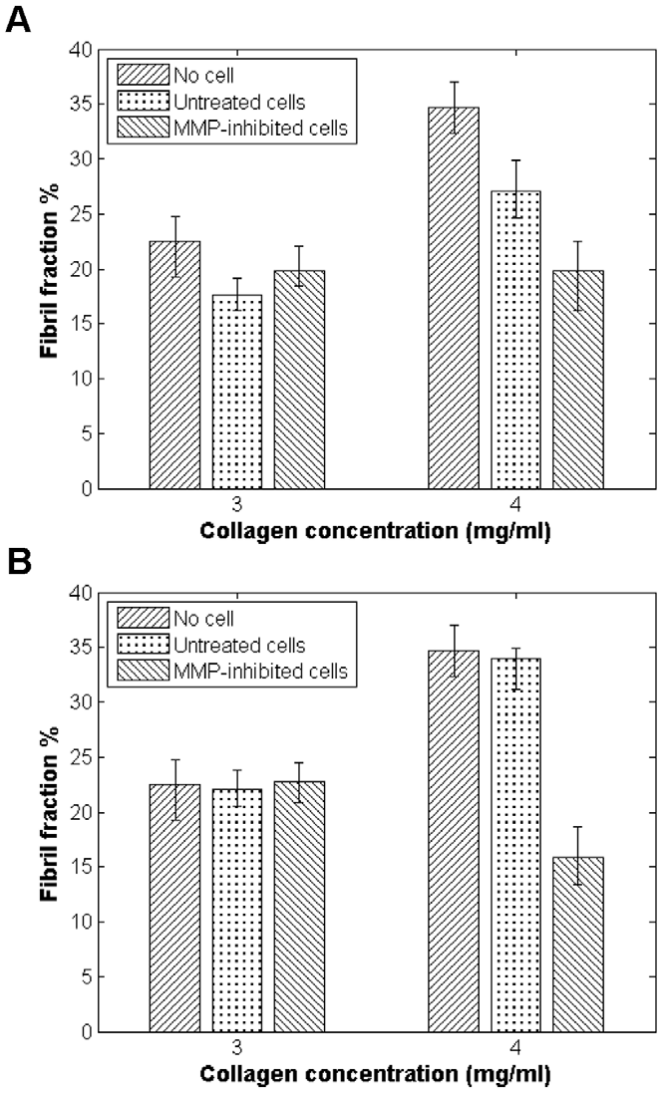
Effect of MMP inhibition on matrix structural properties, as compared to control (no cell condition) and cells not treated by Marimastat. Fibril fractions from cellular regions of gels measured 3 days after seeding for gels seeded with (*A*) LNCaP cells; and (*B*) DU-145 cells.

To test this hypothesis, a broad-spectrum MMP inhibitor, Marimastat, which blocks the activity of MMP-1, 2, 3, 7, 9, and 12 [27], was used to treat cells seeded in collagen gels of varying concentration. Once MMP activity was inhibited in both cell lines, the fibril fractions of the 4 mg/ml gels were much reduced from what was seen in the gels seeded with untreated cells (**Figure 6**), even falling below what was seen in the control gels. This result supports our hypothesis that increased MMP activity may allow for cells to continue to grow and thrive in highly fibrous microenvironments. In the absence of such activity, cells remodel the matrix to better accommodate their modified needs.

## Discussion

Matrix remodeling is a key step in cell migration, invasion and metastasis. A quantitative understanding of how tumor cells at different stages of disease progression alter their microenvironments is critical for a comprehensive understanding of disease progression and therapeutic intervention. Here we have examined how matrix structure evolves over time using quantitative CRM, studying 3D collagen matrices of varying concentration seeded with two prostate cancer cell lines of varying invasiveness.

We found that collagen gels without cells displayed a higher shear modulus and exhibited smaller pore size and higher fibril fraction with increasing collagen concentration as expected. The fibril fraction and pore size of these control gels did not change significantly with time. We also were able to demonstrate that LNCaP cells and DU-145 cells are actively remodeling their environment. After one week, both cell types increased the fibril fraction and reduced the pore size of the 2 mg/ml gels compared to the control. Meanwhile, in 4 mg/ml gels, cells modified the matrices to reduce fibril fraction and increase pore size. The structural changes observed were detectable over a timescale of several days and were found to occur relatively uniformly throughout the matrices, as there was no difference between acellular and cellular regions of LNCaP- or DU-145-seeded gels.

It was also shown that the LNCaP cells and DU-145 cells remodel the matrix in distinct ways, implying that different cell types favor different types of microenvironments and will remodel them accordingly to match their needs. On average, LNCaP cells appeared to favor lower collagen content and higher pore sizes than DU-145 cells. 2 mg/ml collagen gels seeded with LNCaP cells display shorter, narrower fibers on average than their control and DU-145 counterparts. At higher concentrations, the fiber profiles for each collagen concentration for the three conditions (control, LNCaP, and DU-145) were less distinguishable, suggesting that it is the organization of the matrix rather than the structure of the individual collagen fibrils themselves that are different between the different gel conditions.

Upregulated matrix remodeling is a key feature of tumors. Remodeling includes deposition of new ECM, degradation of existing matrix components, and fiber alignment. Deposition of new ECM may not be expected in tumor microenvironments as one might assume that cells in 3D matrices would prefer to deal with fewer steric obstacles to invasion. However, intermediate rather than very low ECM ligand concentrations have been shown to be optimal for cell movement due to the need to balance traction and adhesion forces [28]. Histological studies have indicated that malignant tissues also show increased collagen deposition [29]. Recently it has been demonstrated that invasive cancer cell lines, including LNCaP cells, produce a type I collagen that is resistant to MMP-degradation and facilitates proliferation and migration [30]. In the 2 mg/ml collagen gels especially then, ECM deposition may be important for cell adhesion and movement, explaining the increased collagen content observed in the presence of cells.

Conversely, when cancer cells encounter dense networks of ECM such as the basement membrane, increased matrix proteolysis and fiber alignment becomes critical to cell movement. Prostate cancer biopsies exhibit higher MMP levels than normal tissues [29]. It is possible that DU-145 cells did not remodel the relatively dense 3 and 4 mg/ml collagen matrices to as great an extent as the LNCaP cells since DU-145 cells are more invasive [22]–[24] and express higher levels of MMPs, including MT1-MMP [23], and are consequently more capable of moving through such dense environments. LNCaP cells need to organize the matrix by selectively degrading or aligning fibers using their actomyosin machinery to achieve larger pore sizes. Since collagen can in addition to being an obstacle to migration, facilitate invasion and proliferation [30], DU-145, as the more invasive cell line, is better suited to a more heavily fibrous gel.

When MMPs were inhibited in both cell lines, we find that the cells' preference for denser matrices was concomitantly reduced, as the fibril fractions in 4 mg/ml gels seeded with Marimastat-treated cells were much lower than in gels seeded with untreated cells. The drop was especially dramatic for gels seeded with DU-145 cells, the more invasive cell line. This suggests that active MMPs are a major player in determining how cells remodel the matrix, and that increased MMP activity may not lead simply to reduced ECM content as might be expect. Rather, our data indicates that MMPs can actually result in more fibrous microenvironments. This suggests that MMPs can affect matrix remodeling via alternative pathways besides just degrading the matrix. Indeed, MMPs have been implicated in gel contraction [31], with MMP inhibition resulting in the reduction of collagen gel contraction [32]. Furthermore, it has been shown that MMP inhibition results in decreased collagen synthesis [33], [34], which may also explain the overall reduction in fibril fractions seen in our Marimastat treatment experiments.

The preference of the untreated DU-145 cells for more collagen-rich environments is also consistent with the observation that tissues stiffen during cancer progression [35], [36]. In this study we showed that collagen content correlates well with shear modulus. We therefore note that DU-145-modified environments, which showed higher average fibril fractions, are stiffer on average than the LNCaP-modified matrices. However, it remains unclear if the increased stiffness is a cause of cancer development or is instead an effect of cancer progression. Our data here points towards the latter. This work supports the prevailing theory of the disruption of tensional homeostasis as a major characteristic of the malignant phenotype [35], [37]. It should be pointed out though that in our study, we compared the remodeling effects of only two different cell lines. Far more work comparing the remodeling effects of a broad spectrum of cancer cell lines is necessary to be able to make the general statement that more highly invasive cancers prefer higher density microenvironments. It would also be interesting to see if and how non-cancerous cells (i.e. stem cells or fibroblasts for tissue engineering applications) demonstrate different remodeling activity.

Overall, our results provide a novel quantitative picture of matrix remodeling evolution and dynamics and provide a direct comparison of matrix remodeling for two distinct prostate cancer cell lines. We hope that our results will lead to new lines of investigation on the dynamics of matrix remodeling and future experiments will continue to compare both how the structural and mechanical properties of cell-seeded collagen gels evolve with time using cancerous and non-cancerous cell types. Such an investigation will provide further and desperately needed information on understanding, managing, and combating metastasis.

## Materials and Methods

### Cell culture

Two prostate cancer cell lines, LNCaP and DU-145 (both from ATCC, Manassas, VA), were studied. LNCaP cells were cultured in RPMI-1640 media and DU-145 cells were cultured in F12K media. Both types of media were supplemented with 10% v/v fetal bovine serum (FBS) and 1% v/v penicillin-streptomycin (10,000 IU/mL penicillin; 10,000 µg/mL streptomycin) (all cell culture reagents from ATCC). The cells were maintained at 37°C, 5% CO_2_ in an incubator. Cells were stained with 5 µm CellTracker™ Orange CMRA (Invitrogen, Carlsbad, CA) according to the manufacturer's instructions for cells in suspension prior to collagen seeding. Once seeded in collagen, cells were maintained in antibiotic-free media supplemented with 10% FBS.

### Preparation of collagen gels

Cells were suspended in 3D type I collagen (BD Biosciences, San Jose, CA) at a final density of 200,000 cells/mL. For gels with cells, the collagen matrix solution consisted of LNCaP or DU-145 cells suspended in the appropriate media supplemented by 10% v/v FBS, and an equal volume of collagen and neutralizing solution (100 mM HEPES buffer (Fisher Scientific, Pittsburgh, PA) in 2× phosphate buffered saline (PBS), pH 7.3), as previously described [38]. Gels without any cells were prepared the same way except that the cell suspension volume was replaced with media. Final collagen concentration was varied from 2, 3, and 4 mg/mL. The total volume of each gel solution was 1 mL. The matrix solution was allowed to gel in a 35 mm glass-bottom dish (MatTek, Ashland, MA) at 37°C and 5% CO_2_ in an incubator for 2 hours before 2 mL of 10% v/v FBS-supplemented media was added. Media was replaced every two to three days. Gels were prepared in triplicate for each condition.

### Rheometry

The mechanical properties of gels of varying collagen content (2, 3, 4 mg/mL) were evaluated using rheometry. Briefly, collagen solutions without cells (composition as previously described) were gelled directly on the rheometer (TA Instruments AR2000 Rheometer) at 37°C for 30 minutes. Immediately following gelation, measurements were taken using a cone-shaped geometry, oscillating from 0.1–10 Hz at a torque of 0.1 µN*m. Three samples for each condition were measured. A power law fit was applied to the data to obtain a value for the bulk storage and elastic modulus at 10 Hz.

### Confocal microscopy

To assess the microstructure of the gels, CRM was performed using a scanning confocal microscope (Olympus FV1000) with a 60× 1.2 N.A. water immersion lens. The collagen plated in glass-bottom dishes were excited with a low intensity 488 nm laser and light between 485–495 nm light was collected. Images were acquired at least 100 µm into the gel to avoid edge effects. For the control gels that were not seeded with cells, three 30 µm stacks with 0.5 µm-thick slices were obtained from randomly selected regions in the gel 1, 3, 5, and 7 days after plating. At each time point, for each gel that was seeded with cells, three stacks were taken in regions containing cells and three stacks were taken in regions that did not contain cells (i.e. had no cells within 10 µm above, below, or laterally). Regions with and without cells were identified by performing confocal fluorescence microscopy (CFM) simultaneously with CRM. For CFM, a 543 nm laser was used with the settings for the excitation/emission spectra of Alexa Fluor 546. In cellular regions, a CFM image of the cells was obtained in parallel with the CRM image of the collagen structure. The same microscope settings were used for each acquisition to ensure that results were comparable.

### Image analysis

Raw CRM data was analyzed to obtain collagen structural parameters. The same processing settings were used from image to image to ensure consistency. Fibril fraction and pore size were obtained with ImageJ (NIH, Bethesda, MD). Briefly, 2D images from each stack were binarized, with collagen fibers indicated by black pixels. The binarized images were then used to calculate the fraction occupied by collagen, similar to what has been previously reported [39]. Pore size was measured by drawing three lines (horizontal, vertical, and diagonal, avoiding cells when they were present) across the binarized image in the center of each stack, and using the plot profile function in ImageJ. A script was written in Matlab (MathWorks, Natick, MA) to calculate the distance between collagen fibers from this profile data. This again is similar to what has been performed before [40].

Fiber diameter and length was determined using Imaris (Bitplane, St. Paul, MN). A rough surface mask was initially made from the raw CRM data, from which a smooth surface was generated. The objects created with the resulting smooth surface each represented a collagen fibril and statistics on the radius and half length of each fibril were output.

### Quantitative gelatin zymography

Gelatin zymography was utilized to compare MMP activity between cells lines. Both LNCaP and DU-145 cells were plated and grown to near confluency before incubation with serum-free media for 24 hours. Media was extracted from cultures, and concentrated via ultracentrifugation for 30 min (10 kDa cutoff). Samples were then mixed with Laemmli loading buffer without a reducing agent and subjected to gelatin zymography as previously described [41]. Briefly, samples were loaded into a polyacrylamide gel co-polymerized with 0.1% gelatin and subjected to electrophoresis. Gels were then transferred to an aqueous solution containing 2.5% Triton-X100 to renature the proteins, followed by equilibration in a developing buffer (50 mM Tris, pH 7.8, 200 mM NaCl, 5 mM CaCl_2_, 0.02% Brij-35) and subsequent incubation at 37°C for 20 hrs. Gels were finally stained and destained with Coomassie blue. After destaining, gels were dried overnight using a gel drying kit (Promega, Madison, WI). Areas of proteolytic activity are expressed as clear bands against a stained background. Gel images were processed with ImageJ and thresholded to acquire appropriate pixel values for both MMP-2 and MMP-9 followed with normalization by original cell concentrations. The experiment was performed in triplicate.

### MMP inhibition studies

Cells were seeded in gels of varying collagen content (2, 3, and 4 mg/ml) in 12 well glass-bottom plates (MatTek). The total volume of each of these smaller gels was 0.5 ml. Each gel was treated with 50 µM Marimastat (Tocris, Ellisville, MO) in 1 mL of 10% FBS-supplemented media 2 hours after initial gelation and being placed in the incubator. Thereafter, the media was replaced with 100 µM Marimastat in 10% FBS-supplemented media every 24 hours. Gels were prepared in triplicate. CFM and CRM was performed 1 and 3 days after seeding. Image processing was performed as described earlier.

### Statistical analysis

Since none of the CRM structural data followed normal distributions as assessed by q-q plots, 95% confidence intervals were constructed using bootstrapping from at least 5,000 simulations. The bias-corrected, accelerated algorithm was used. Analysis was performed with Matlab. Error bars on all graphs are 95% confidence intervals unless otherwise noted.

## Supporting Information

Figure S1 **Structural parameters for gels without cells.** (*A*) Fibril fraction and (*B*) pore size over time for all three gel concentrations. (*C*) Fiber diameters and (*D*) fiber lengths for 2 mg/ml collagen over time. (*E*) Fiber diameters and (*F*) fiber lengths at day 7 for all gel concentrations.
(TIF)

Figure S2 **Structural parameters for the cellular regions of DU-145 cells and LNCaP cells compared to the no cell condition over time.** Fibril fraction for (*A*) 2 mg/ml; (*C*) 3 mg/ml; and (*E*) 4 mg/ml. Pore size for (*B*) 2 mg/ml; (*D*) 3 mg/ml; and (*F*) 4 mg/ml.
(TIF)
