# Supplementary figures and images for: Quantitative Analysis of the Effect of Cancer Invasiveness and Collagen Concentration on 3D Matrix Remodeling
Source: PLoS One. 2011 Sep 27;6(9):e24891. doi: 10.1371/journal.pone.0024891 (PMC3181246; doi:10.1371/journal.pone.0024891)

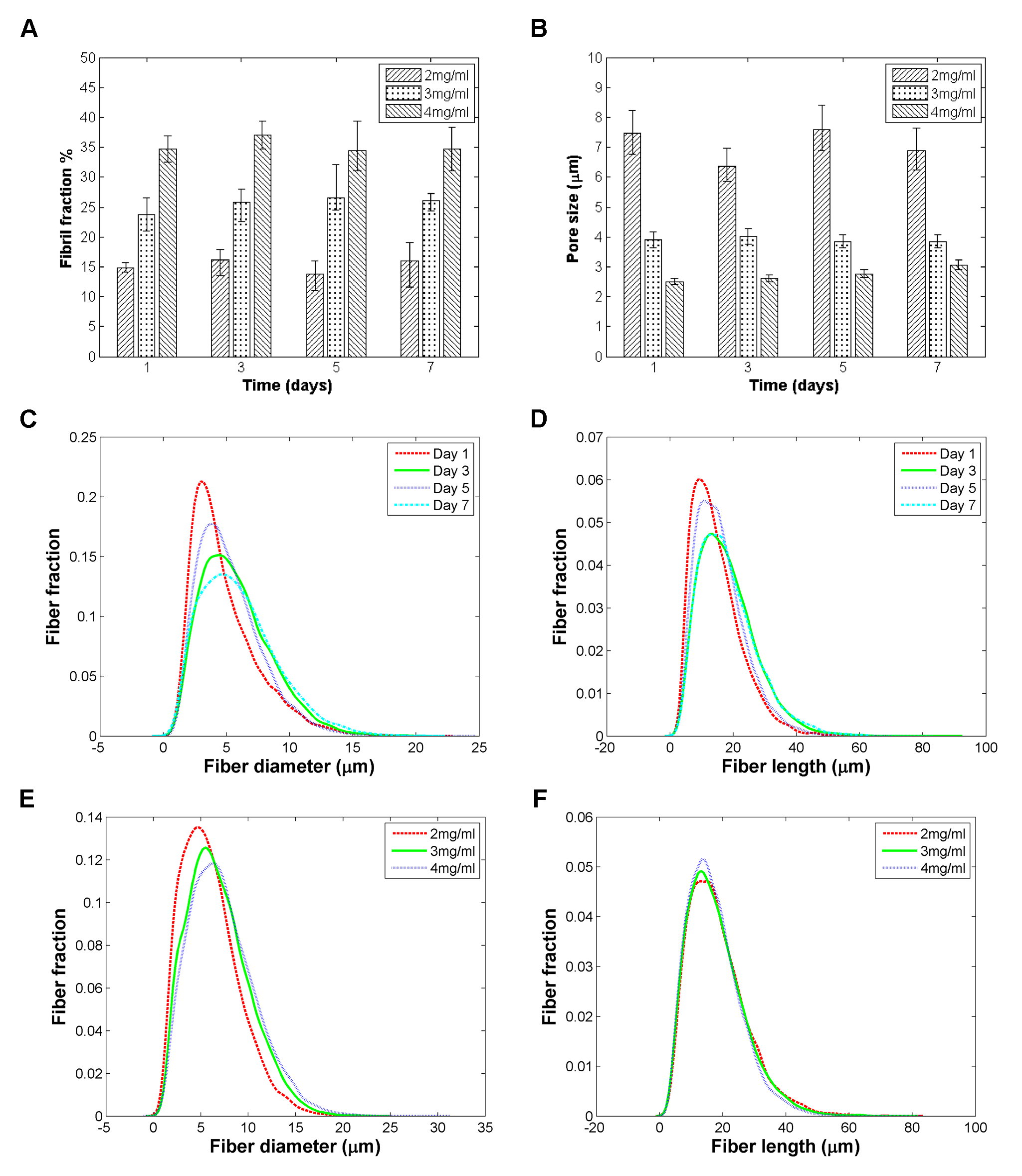

Supplement: Figure S1 — Structural parameters for gels without cells. (A) Fibril fraction and (B) pore size over time for all three gel concentrations. (C) Fiber diameters and (D) fiber lengths for 2 mg/ml collagen over time. (E) Fiber diameters and (F) fiber lengths at day 7 for all gel concentrations. (TIF) [file pone.0024891.s001.tif]

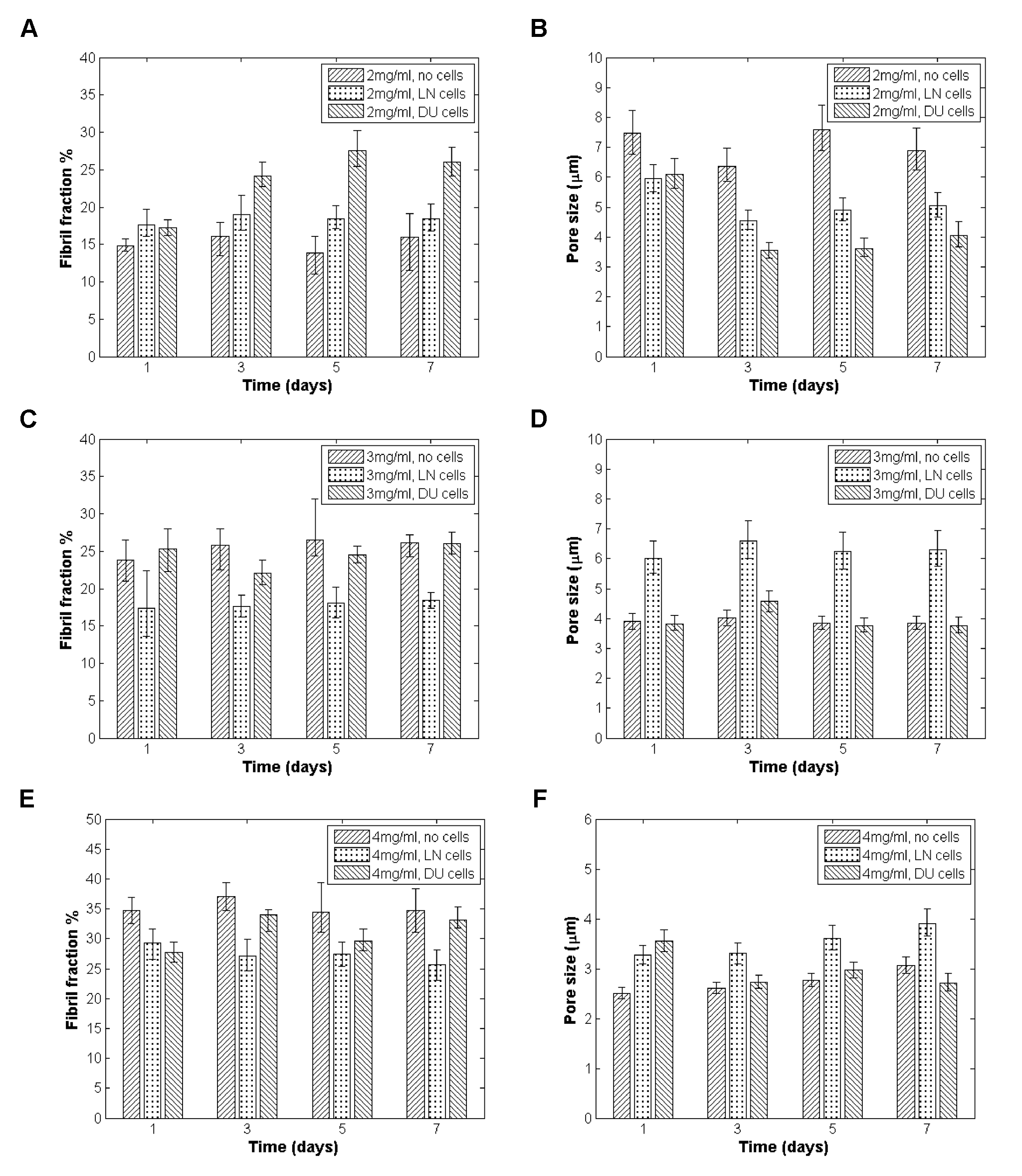

Supplement: Figure S2 — Structural parameters for the cellular regions of DU-145 cells and LNCaP cells compared to the no cell condition over time. Fibril fraction for (A) 2 mg/ml; (C) 3 mg/ml; and (E) 4 mg/ml. Pore size for (B) 2 mg/ml; (D) 3 mg/ml; and (F) 4 mg/ml. (TIF) [file pone.0024891.s002.tif]
